# Causative variants linked with limb girdle muscular dystrophy in an Iranian population: 6 novel variants

**DOI:** 10.1002/mgg3.2101

**Published:** 2022-11-14

**Authors:** Hamidreza Mianesaz, Safoura Ghalamkari, Mansoor Salehi, Mahdiyeh Behnam, Majid Hosseinzadeh, Keivan Basiri, Majid Ghasemi, Maryam Sedghi, Behnaz Ansari

**Affiliations:** ^1^ Department of Human Genetics, Medical School University of Debrecen Debrecen Hungary; ^2^ Department of Genetics and Molecular Biology Isfahan University of Medical Sciences Isfahan Iran; ^3^ Division of Clinical Genetics, Department of Laboratory Medicine, Faculty of Medicine University of Debrecen Debrecen Hungary; ^4^ Cellular, Molecular and Genetics Research Center Isfahan University of Medical Sciences Isfahan Iran; ^5^ Student Research Committee Semnan University of Medical Science Semnan Iran; ^6^ Medical Genetics Laboratory, Alzahra University Hospital Isfahan University of Medical Sciences Isfahan Iran; ^7^ Metabolic Disorders Research Center, Endocrinology and Metabolism Molecular‐Cellular Sciences Institute Tehran University of Medical Science Tehran Iran; ^8^ Department of Neurology Isfahan University of Medical Sciences Isfahan Iran; ^9^ Isfahan Neuroscience Research Center, ALzahra Research Institute Isfahan University of Medical Science Isfahan Iran

**Keywords:** calpain, dysferlin, laminin, LGMD, limb‐girdle muscular dystrophy, muscular disorders, sarcoglycans, whole exome sequencing

## Abstract

**Background:**

Limb‐girdle muscular dystrophy (LGMD) is a non‐syndromic muscular dystrophy caused by variations in the genes involved in muscle structure, function and repair. The heterogeneity in the severity, progression, age of onset, and causative genes makes next‐generation sequencing (NGS) a necessary approach for the proper diagnosis of LGMD.

**Methods:**

In this article, 26 Iranian patients with LGMD criteria were diagnosed with disease variants in the genes encoding calpain3, dysferlin, sarcoglycans and Laminin α‐2. Patients were referred to the hospital with variable distribution of muscle wasting and progressive weakness in the body. The symptoms along with biochemical and EMG tests were suggestive of LGMD; thus the genomic DNA of patients were investigated by whole‐exome sequencing including flanking intronic regions. The target genes were explored for the disease‐causing variants. Moreover, the consequence of the amino acid alterations on proteins' secondary structure and function was investigated for a better understanding of the pathogenicity of variants. Variants were sorted based on the genomic region, type and clinical significance.

**Results:**

In a comprehensive investigation of previous clinical records, 6 variations were determined as novel, including c.1354–2 A > T and c.3169_3172dupCGGC in *DYSF*, c.568 G > T in *SGCD*, c.7243 C > T, c.8662_8663 insT and c. 4397G > C in *LAMA2*. Some of the detected variants were located in functional domains and/or near to the post‐translational modification sites, altering or removing highly conserved regions of amino acid sequence.

## Graphical abstract

Causative variants linked with LGMD in an Iranian population.

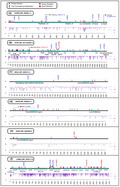

## 1 INTRODUCTION

Among the proteins involved in myocyte function, dystrophin‐glycoprotein complex (DGC) is a large multi‐component protein complex uniquely expressed in the skeletal and cardiac muscle membrane. DGC supplies the coherence between the myofibrils, myocytes membrane and extracellular matrix (ECM) (Gawor & Prószyński, 2018). Dystrophin is the most known protein component working as the first line protein of DGC. Due to its significantly large size, dystrophin is highly susceptible to sporadic variants. Duchene or Becker (DMD or BMD), the most prevalent muscular dystrophies, arises from variants in the coding sequence of dystrophin. Other lower penetrance and hereditary muscular disorders called Limb‐Girdle Muscular Dystrophy (LGMD) exist with almost similar but milder clinical features compared to that of DMD (Lapidos et al., 2004). LGMD demonstrates autosomal dominant and recessive inheritance shown by LGMD1 (AD‐LGMD) and LGMD2 (AR‐LGMD) respectively. Clinical features of LGMD vary slightly depending on the mutant allele and include muscle pain, swelling, weakness and wasting along with calf hypertrophy and scapular winging; it seldomly coincides with cardiomyopathy (Walton & Nattrass, 1954). The heterogeneity of LGMD symptoms and severity partly originates from the compensation by the other variants which are expressed more in other tissues and it makes the diagnosis more complicated (Wheeler et al., 2004).

Variants in sarcoglycans subunits, a group of DGC components encoded by *SGCA* (OMIM: 600119), *SGCB* (OMIM: 600900), *SGCD* (OMIM: 601411), *SGCG* (OMIM: 608896), *SGCE* (OMIM: 604149) and *SGCZ* (OMIM: 608113), can lead to different types of LGMD. In the absence of any subunit, the sarcoglycan complex assembly in the endoplasmic reticulum wents defective or is reduced significantly (Hack et al., 1998). Although many known types of LGMD arise from variants in DGC components, a group of LGMD occurs due to variants in genes involved in muscle regeneration and function e.g., calpain3, dysferlin, titin, caveolin etc. (Murphy & Straub, 2015). LGMD2A is one the most common forms of LGMD caused by variants in the *CAPN3* (OMIM: 114240) (Sorimachi et al., 1989). The calpain3 is a muscle‐specific cysteine protease that requires calcium for its proper function. Although the exact pathogenesis of LGMD2A is not well‐understood, several mechanisms have been suggested so far (Huang et al., 2008). The dysferlin and caveolin are associated with LGMD2B and LGMD1C respectively, localized in the membrane apart from DGC and are involved in membrane assembly and repair. The dysferlin is encoded by the *DYSF* (OMIM: 603009) and plays also an important role in muscle fiber integrity and repair (Bashir et al., 1998).

Laminin, the major non_collagenous component of basement membrane, is a heterotrimeric molecule consisting of 3 different subunits (α, β and γ); each one encoded by various genes in the human genome (Hohenester, 2019a). Each laminin forms a tertiary structure consisting of one long arm and three short arms which are capable of binding to the cells and other laminin molecules respectively (Beck et al., 1990). Depending on the composition of different subunits, 15 different chain combinations have been discovered so far (Aumailley et al., 2005; Hohenester, 2019b). Laminin‐α2 or merosin is encoded by *LAMA2* (OMIM: 156225) and takes part in the formation of laminin 211 (Oliveira et al., 2018). Merosin along with other components can bind to the glycosylated residues of alpha‐dystroglycan (DAG1) which is an important DCG component. Variants in *LAMA2* mainly cause severe congenital muscular dystrophy (CMD type 1A) called Merosin deficiency (Helbling‐Leclerc et al., 1995; Oliveira et al., 2008). However, it can also associate with milder symptoms with later onset muscular dystrophy called LGMD(R)23 (Chan et al., 2014; Gavassini et al., 2011).

Efficient genetic diagnosis is indispensable for proper genetic consoling and treatment of LGMD patients. Due to a broad variant spectrum causing this disorder, next‐generation sequencing (NGS) is one of the best suggestions for the diagnosis of LGMD. In this study, molecular genetic changes in a set of patients who had strong criteria of limb‐girdle muscular dystrophy were investigated by using whole‐exome sequencing (Chou et al., 1999).

## 2 RESULTS

### 2.1 Calpainopathies

Eight known variants were found in *CAPN3* causing LGMD2A (Table 1). Two probands were diagnosed with 1 base pair (bp) c.2256_2257insA insertion in exon 21 causing frameshift as well as a predicted effect on the splice site (8 bp distance). These patients were wheelchair‐bound brothers (19 and 21 years old) presenting LGMD symptoms including weakness and wasting of proximal muscles from age of 13. This variant has been previously reported in 2 cases in a broad study on 1001 patients with unexplained limb‐girdle weakness (Töpf et al., 2020). Another proband was detected with c.259 C > G variant in exon 1 of *CAPN3*. The segregation study showed the father and other unaffected siblings with the same variant in heterozygous forms. This variant has been firstly reported in 2005 in a study mainly focused on the European (Italian) population (Piluso et al., 2005). In another proband, two variants were found in compound heterozygous alleles in *CAPN3*. These variants included c.2105 C > T in exon 19 and c.2380 + 2 T > G in intron 22 of *CAPN3* with 2 bp distance from the splice site. The first allele with c.2105 C > T variant has been previously reported in 2 different studies (Fattahi et al., 2017; Mojbafan et al., 2018) as well as one record for c.2380 + 2 T > G (Fadaee et al., 2016); all in the Iranian population. In another proband, 2 known variants were found suggesting compound heterozygous mode. These variants which were previously reported in two separate studies are c.145 C > T in exon 1 (Yu et al., 2017) and c.956 C > T in exon 7 (van der Kooi et al., 2007) reported in Chinese and Nederlands populations respectively. The c.145 C > T variant frequency is extremely low in the general population according to genomAD. Moreover, another pathogenic variant in which the same amino acid is changed to histidine (rs863224958) has been reported by several other submitters. On the other hand, c.956 C > T is also an extremely low frequent allele in the population and several pathogenic variants have been reported in the very close amino acids. The c.946–2 A > G is a formerly reported variant (Groen et al., 2007; Park et al., 2017; Richard et al., 1999) in intron 6 of *CAPN3* which was detected in two probands from unrelated families in our study. This variant has a 2 bp distance from the splice site. A segregation study in one of the families revealed that parents were heterozygous for this variant. Another proband was recorded with c.1469G > A homozygous variant in exon 10 of the *CAPN3* which also has been previously reported (Dinçer et al., 1997; Fanin et al., 2003). A pathogenic variant in which the same amino acid is changed to tryptophan (rs141656719) has been reported by a high number of publications and reports.

**TABLE 1:** Variant spectrum in *CAPN3* (NG_008660.1) encoding calpain3 associated with the patients diagnosed with limb girdle muscular dystrophy or calpainopathy

Variant #	Proband count	Gene	Position on cDNA	Exon/ intron	Type of variant	Position on protein	Splice site/distance (nt)	Alleles	Pathogenicity (ACMG)	Reference SNP	Heterozygous allele in family	Novel variant	Ref
1	2	*CAPN3*/15q15.1	c.2256_2257 insA	Exon 21	Frameshift	p.D753fs	**+/8**	Homozygous	PVS1	–	–	No	Töpf et al. (2020)
2	1	*CAPN3*	c.259 C > G	Exon 1	Missense	p.L87V	–	Homozygous	PP1	rs558925493	p,s	No	Piluso et al. (2005)
3	1	*CAPN3*	c.2105 C > T	Exon 19	Missense	p.A702V	**+/10**	Compound heterozygous	PM1/PP3	–	s	No	Fattahi et al. (2017); Mojbafan et al. (2018)
4			c.2380 + 2 T > G	Intron 22	Splice site	–	**+/2**		PVS1	rs761935462	s	No	Fadaee et al. (2016)
5	1	*CAPN3*	c.145 C > T	Exon 1	Missense	p.R49C	–	Compound heterozygous	PM2	rs794726871	–	No	Yu et al. (2017)
6			c.956 C > T	Exon 7	Missense	p.P319L	–		PM1/PM2	rs121434547	–	No	van der Kooi et al. (2007)
7	2	*CAPN3*	c.946–2 A > G	Intron 6	Splice site	–	**+/2**	Homozygous	PVS1	rs1595826673	p,m	No	Groen et al. (2007); Park et al. (2017); Richard et al. (1999)
8	1	*CAPN3*	c.1469 G > A	Exon 10	Missense	p.R490Q	–	Homozygous	PM1	rs121434548	–	No	Dinçer et al. (1997); Fanin et al. (2003)

Abbreviations: m, maternal; p, paternal; PM, pathogenic moderate; PP, pathogenic possible; PS, pathogenic strong; PVS, pathogenic very strong; s, siblings.

### 2.2 Dysferlinopathies

Six variants were detected in the *DYSF* causing LGMD type 2B (Table 2). A novel pathogenic variant c.1354–2 A > T was detected in intron 14 of *DYSF*. This variant has a 2 bp distance from the splice site and therefore it is predicted to affect the splicing of the intron 14. The segregation study in this family showed father and mother were heterozygous for the variant and among the other 2 siblings, one was a heterozygous carrier and the other one was non‐mutated homozygous. Another proband was homozygous for a known variant c.3166C > T in exon 28 of the *DYSF* (Nguyen et al., 2005; Takahashi et al., 2013). In a patient with LGMD criteria, c.5921C > T missense variant was detected in exon 53 of DYSF. Up to the current date, this variant has been reported with LGMD criteria in a compound heterozygous form (rs1573176526) (National Center for Biotechnology Information, 2020). This variant was absent from large population studies to date (see discussion). In a patient, a homozygous c.2760dupC frameshift variant was found in exon 25 of the *DYSF* which was previously recorded in Clinvar as pathogenic. Also, c. 6155 C > G homozygous variant in exon 54 of *DYSF* was found in a proband to be associated with the LGMD criteria. This variant is reported in homozygous form in an LGMD patient for the first time in this study (see discussion) and the allele is reported at extremely low frequency in large population studies (only 1 was allele reported in the East Asian population). Although the c. 6155 C > G variant has been associated with a reference number (rs886044643), it has only one record in heterozygous form with which no criteria or interpretation has been recorded up to the date of this publication. Another novel 3169_3172dupCGGC homozygous variant was detected in an LGMD patient which was confirmed by segregation analysis and sanger sequencing. A missense variant in a similar position (c.3172C > T) has been submitted as “Likely Pathogenic” and has been reported in 3 separate publications (Walter et al., 2013).

**TABLE 2:** Variant spectrum in *DYSF* (NG_008694.1) encoding dysferlin associated with the patients diagnosed with limb girdle muscular dystrophy (dysferlinopathy)

Variant #	Proband count	Gene	Position on cDNA	Exon/ intron	Type of variant	Position on protein	Splice site/distance (nt)	Alleles	Pathogenicity (ACMG)	Reference SNP	Heterozygous allele in family	Novel variant	Ref
1	1	*DYSF*/2p13.2	c.1354–2 A > T	Intron 14	Splice site	–	+/2	Homozygous	PVS1	–	**p,m,s**	**Yes**	–
2	1	*DYSF*	c.3166 C > T	Exon 28	Nonsense	p.R1056X	–	Homozygous	PVS1	rs369607332	–	No	Takahashi et al. (2013); Nguyen et al. (2005)
3	1	*DYSF*	c.5921 C > T	Exon 53	Missense	p.P1974L	–	Homozygous	PM2	rs1573176526	–	No	–
4	1	*DYSF*	c.2760dupC	Exon 25	Frameshift	p.K921fs	–	Homozygous	PVS1	rs1559177278	–	No	–
5	1	*DYSF*	c.6155 C > G	Exon 54	Missense	p.P2052R	–	Homozygous	PM2	rs886044643	–	No	–
6	1	*DYSF*	c.3169_3172dupCGGC	Exon 29	Frameshift	p.R1058fs	–	Homozygous	PVS1	–	–	**Yes**	–

Abbreviations: m, maternal; p, paternal; PM, pathogenic moderate; PP, pathogenic possible; PS, pathogenic strong; PVS, pathogenic very strong; s, siblings.

### 2.3 Sarcoglycanopathies

In this study, 5 variants were reported in sarcoglycan genes (Table 3). A proband was discovered with c.221G > A variant in exon 3 of the *SGCA* causing LGMD2D. There are more pieces of evidence already recorded for this variant in ClinVar. Of note, in other available likely pathogenic/pathogenic variant records, although the nucleotide 221G is not necessarily affected, the same amino acid residue (Arg) has been changed which can confirm the pathogenicity of this variant. Another proband was diagnosed with a known nonsense variant c.574C > T in exon 5 of *SGCA* leading to LGMD2D. This variant has been reported as the first identified case of alpha‐sarcoglycanopathy in Albania (Babameto‐Laku et al., 2011). A proband with LGMD2F was found to harbor c.568 G > T novel nonsense variation in exon 7 of *SGCD*. This variant has an 8 bp distance from the splice site and might have an adverse effect on splicing. A known variant c.275 T > C in exon 3 of the *SGCB* was detected in a patient with LGMD2E. This variant was only reported once in the population of Turkey (Balci et al., 2004); however, this allele was not available in large population studies. Two unrelated patients were also homozygous for an Exon2 deletion (c.34_243del) in *SGCB* which is an already known variant causing LGMD2E. This allele was confirmed by gap‐PCR in both probands. This variant has been previously reported in 3 different pieces of literature on the Iranian population (Ghafouri‐Fard et al., 2017; Mojbafan et al., 2020; Taghizadeh et al., 2018).

**TABLE 3:** Variant spectrum in different genes encoding sarcoglycan compartments (SGCA: NG_008889.1, SGCB: NG_008891.1, SGCD: NG_008693.2) associated with the patients diagnosed with limb girdle muscular dystrophy (Sarcoglycanopathy)

Variant #	Proband count	Gene	Position on cDNA	Exon/ intron	Type of variant	Position on protein	Splice site/distance (nt)	Alleles	Pathogenicity (ACMG)	Reference SNP	Novel variant	Ref
1	1	*SGCA/* 17q21.33	c.221 G > A	Exon 3	Missense	p.R74Q	–	Homozygous	PM1/PM5	rs779439298	No	–
2	1	*SGCA*	c.574 C > T	Exon 5	Nonsense	p.R192X	–	Homozygous	PVS1	rs387907298	No	Babameto‐Laku et al. (2011)
3	1	*SGCD*/ 5q33.2‐q33.3	c.568 G > T	Exon 7	Nonsense	p.E190X	+/8	Homozygous	PVS1	–	**Yes**	–
4	1	*SGCB*/ 4q12	c.275 T > C	Exon 3	Missense	p.I 92 T	–	Homozygous	PM2	–	No	Balci et al. (2004)
5	2	*SGCB*	Exon2 Deletion c.34_243del	Exon 2	Frameshift	p.Q12fs	–	Homozygous	PVS1	–	No	Ghafouri‐Fard et al. (2017); Mojbafan et al. (2020); Taghizadeh et al. (2018)

Abbreviations: PM, pathogenic moderate; PP, pathogenic possible; PS, pathogenic strong; PVS, pathogenic very strong.

### 2.4 *LAMA2* variants

In this study, 6 variants have been detected in *LAMA2* with an interpretation of LGMDR23 in patients (Table 4). A novel nonsense disease‐causing variant in c.7243C > T was found in exon51 of *LAMA2* in a patient with LGMD symptoms. A known homozygous variant of c.1303 C > T on exon 9 of *LAMA2* was found in a patient. This nonsense variant has a 4 bp distance from the splice site and has been reported in several populations so far (Geranmayeh et al., 2010; Xiong et al., 2015). In a proband, a novel homozygous insertion c.8662_8663insT was found in exon 61 of *LAMA2* which results in a frameshift in the output amino acid chain. A known homozygous c.4035 T > G variant in exon 27 was found in an LGMD proband which has been previously reported in the European population (Geranmayeh et al., 2010). An exon 27 variant of c. 3976 C > T was found in an LGMD proband which has been previously reported in a patient with CMD1A criteria in the European population (Oliveira et al., 2008). A novel disease‐causing c. 4397G > C variant in an LGMD proband was also discovered in exon 30 of *LAMA2*. This variant was absent in controls in large population studies.

**TABLE 4:** The variant spectrum in *LAMA2* (NG_008678.1) encoding laminin α‐2 protein, associated with the patients diagnosed with limb girdle muscular dystrophy

Variant #	Proband count	Gene	Position on cDNA	Exon/ intron	Type of variant	Position on protein	Splice site/distance (nt)	Alleles	Pathogenicity (ACMG)	Reference SNP	Novel variant	Ref
1	1	*LAMA2* 6q22.33	c.7243 C > T	Exon 51	Nonsense	p.Q2415X	–	Homozygous	PVS1	–	**Yes**	–
2	1	*LAMA2*	c.1303 C > T	Exon 9	Nonsense	p.R435X	+/4	Homozygous	PVS1	rs773209126	No	Xiong et al. (2015); Geranmayeh et al. (2010)
3	1	*LAMA2*	c.8662_8663 insT	Exon 61	Frameshift	p.G2888fs	–	Homozygous	PVS1	–	**Yes**	–
4	1	*LAMA2*	c.4035 T > G	Exon 27	Nonsense	p.Y1345X	–	Homozygous	PVS1	–	No	Geranmayeh et al. (2010)
5	1	*LAMA2*	c.3976 C > T	Exon 27	Nonsense	p.R1326 X	–	Homozygous	PVS1	rs398123373	No	Oliveira et al. (2008)
6	1	LAMA2	c.4397G > C	Exon 30	Missense	p.C1466S	–	Homozygous	PM2	–	**Yes**	–

Abbreviations: PM, pathogenic moderate; PP, pathogenic possible; PS, pathogenic strong; PVS, pathogenic very strong.

## 3 DISCUSSION

**FIGURE 1:**
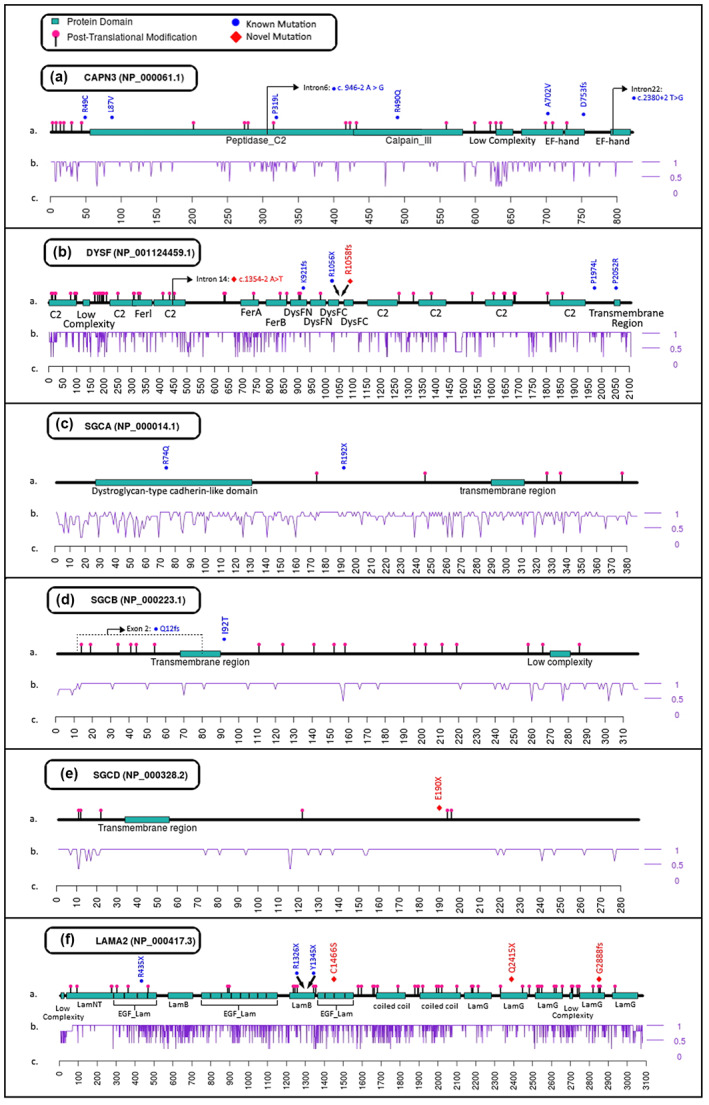
The position of variants are represented in the secondary structure of calpain3 (a), dysferlin (b), sarcoglycan alpha (c), sarcoglycan beta (d), sarcoglycan delta (e) and laminin subunit alpha 2 (f). The position of variants are demonstrated based on the altered amino acid number in the peptide sequence above the first track. The first (top) track in each panel shows the distribution of the variants concerning the approximate position of the known functional domains and post‐translation modification sites. The second track in each panel highlights the conservation score of each amino acid in a range of 0 (lowest) to 1 (highest) and the third track in each panel demonstrates the amino acid count from N to C terminus of the protein.

By molecular genetics investigation of patients with LGMD criteria, we conducted 26 LGMD probands of which 24 were homozygous for the suggested causative variant and 2 of them were compound heterozygous. We detected 25 different variants of which 8 (32%) were in *CAPN3*, 6 (24%) in *DYSF*, 5 (20%) in sarcoglycan genes including 2 variants in *SGCA*, 2 in *SGCB* and 1 in *SGCD*, and 6 (24%) variants in the *LAMA2*. Among these variants, 19(76%) have been either clinically described or available in large population studies in heterozygous form and 6(24%) were novel. Overall, none of the variants in *CAPN3*, %33.3 of variants in *DYSF*, 20% of variants in sarcoglycan genes and 50% of the diagnosed variants in *LAMA2* were classified as novel. Among all variants, 3(12%) have been detected in intronic regions close to the splice sites and 22(%88) were detected in exons. Moreover, among the exon variants, 4(18%) were predicted to affect splicing due to their proximity to the splice site as well as their effect on the amino acid sequence. The position of the variants has been plotted on the secondary structure of the corresponding proteins to shed light on their possible effect on the protein structure and function. Figure 1 shows a visual presentation based on 3 critical criteria of proteins: functional domains, post‐translation modification sites (PTM) and evolutionary conservation score, and demonstrates the position of each variation in this matrix which could help a better understanding of the pathogenicity of the variants.

### 3.1 Calpain3

The majority of the detected variants found in calpain3 are located in highly conserved regions (Figure 1a) highlighting the importance of the altered amino acids in the protein function. Three known individual variants were occurred in the peptidase_C2 domain including a variant at the beginning of Intron 6, potentially affecting the correct mRNA splicing. Interestingly, three variants in calpain3 occurred in the EF‐hand domains located at the C terminal of the protein. EF‐hand domains play an important role in recruiting calcium ions and mediating their role in the signaling as well as calpain III domain function (Gifford et al., 2007). Although calpain III domain function is not still completely known, calcium‐dependent cysteine protease activity has been suggested as one of its roles (Duguez et al., 2006). Regarding the c.2256_2257insA (D753fs) variant detected in calpain3, the frameshift can cause a 12 amino acid change leading to a termination codon causing deletion of 3 last exons. This not only affects the second EF‐hand domain of the protein but also removes the last EF‐hand leading to a truncated protein. In another scenario for the pathogenicity of this variant, it could also contribute to modified splicing due to its proximity to the splice site (Table 1) and can have a different effect on the protein structure by impaired splicing. The A702V variant affects the first EF‐hand domain. It is located in close to PTM sites and also has 10 bp distance from the splice site.

### 3.2 Dysferlin

A number of the detected variants in dysferlin are located in locations known as functional domains (Figure 1b) in highly conserved regions and some of them are adjacent to PTM sites owing to a possible effect on the post‐translation modification (Krassowski et al., 2018). Although the known R1056X and the novel R1058fs adjacent variants are not located in a functional domain, the sequence alteration is quite detrimental due to termination and frameshift effects respectively. Regarding the c.5921C > T (P1974L) missense variant in *DYSF*, although no citation was found for this variant, a patient with LGMD2B harboring this variant in compound heterozygous form has been submitted as a shred of evidence in ClinVar (National Center for Biotechnology Information, 2020). This variant is absent from large population studies and there is no strong support for the impact of this variant on the protein. This is consistent with our study revealing this variant in a region comprising no predicted domains so far. However, the current paper is the first reported case harboring this variant in homozygous form and can be another evidence of its pathogenicity. Of note, two variants were clinically reported for the first time with LGMD criteria in the current paper, namely, c.2760dupC (K921fs) and c.6155C > G (P2052R) in *DYSF*. Although few pieces of evidence were submitted on the pathogenicity of the aforementioned variants in ClinVar, no citation or clinical interpretation has been submitted up to this time. The c.2760dupC variant has been recorded by three submitters as pathogenic but none of them reported the LGMD as the clinical criteria up to this time. However a submission in an Iranian population (Kariminejad ‐ Najmabadi Pathology & Genetics Center) has interpreted “Abnormality of the musculature” as the resultant condition of this variant.

### 3.3 Sarcoglycans

In comparison to the other genes discussed in this paper, sarcoglycans encode shorter and less complex proteins in terms of structure and number of active domains (Figure 1c,e). Two known variants have been detected in alpha sarcoglycan in LGMD patients (Figure 1c). The c.221G > A (R74Q) variant in *SGCA* is located in the middle of the dystroglycan‐type cadherin‐like domain (DGCD) of the protein. Among the previously submitted cases for this variation, a record of an LGMD patient in the Iranian population is available (Nilou‐Genome Lab, 2021). Although this variant is classified with uncertain significance; the presence of several records for this variant along with the LGMD phenotype suggests the association of that with the pathogenesis of LGMD. The other variant in *SGCA* is a nonsense variant leading to a truncated unfunctional protein, although it is not localized in a known active domain. The exon 2 deletion in beta sarcoglycan (Figure 1d) leads to the elimination of codons 12 to 81 comprising a large part of the cytoplasmic and transmembrane regions and numerous important PTM sites. The other detected variant in *SGCB*, I92T is located close to the transmembrane region most probably affecting the function of this domain. The only variant in delta sarcoglycan (Figure 1e) was E190X which is not located in an active domain but can lead to the removal of a large, well‐conserved part of the protein including PTM sites.

### 3.4 Laminin α‐2

Laminin α‐2 is a large protein consisting of several functional domains, PTM sites and highly conserved cysteine residues necessary for its short arms formation as well as trimerization of the subunits (Beck et al., 1990). Except for the C1466S novel variant, all other *LAMA2* variants in our LGMD cases were either frameshift or noncoding variants resulting in pre‐maturation stopped translation (Figure 1f). Interestingly, no polymorphism or variant for missense C1466S variant in large population studies is recorded and no criteria is reported at dbSNP and ensemble. The affected amino acid is among eight extremely conserved cysteine residues in consecutive repeats of about 60 amino acids called laminin‐type epidermal growth factor‐like domains (EGF‐Lam or LE domain) (Engel, 1989). These cysteine residues play an important role in stabilizing rode‐like regions in short arms of laminin through disulfide bond establishment (Beck et al., 1990).

The novel Q2415X and G2888fs variants are located in the globular region of the C‐terminus of laminin α‐2 (Tisi et al., 2000). This region is essential for the interaction between laminin and the specific O‐mannose‐linked glycan modification of the alpha subunit of dystroglycan (α‐DG) transmembrane protein. The proper interaction between these two proteins is essential for muscle function. The first three out of 5 G domains (LamG or LG1 to 5) form a specific cloverleaf structure linked to LG4 and 5 via a flexible region (low complexity domain in Figure 1f) and the LG4 plays the central role in the interaction with α‐DG through the Arg2803 residue. The Q2415X nonsense variant occurs in the LG2 domain of the globular region and results in the elimination of LG3‐5 as well as a large part of LG2. The G2888fs variant though is placed in a PTM‐rich region of the LG4 domain and leads to a frameshift in the amino acid chain. Although the α‐DG binding site (Arg2803 residue) is located before the G2888fs variant, other residues of LG4 and LG5 can play an important role in reducing the electrostatic repulsion between laminin and α‐DG (Hohenester, 2019a) and maintaining the functional structure of the globular region. The two known variants of R1326X and Y1345X are noncoding variants leading to the elimination of a large part of the protein. Besides these variants are located in the second globular domain (LamB) of the short arm of laminin α‐2, which plays an important role in mediating laminin polymerization (Hohenester, 2019b).

Among all diagnosed variants in this study, only 5 variants (20%) have been previously reported in the Iranian population including c.2105 C > T (Fattahi et al., 2017) and c.2380 + 2 T > G in *CAPN3* (Fadaee et al., 2016), c.34_243del exon2 deletion in *SGCB* (Ghafouri‐Fard et al., 2017; Mojbafan et al., 2020; Taghizadeh et al., 2018) and 2 evidence submissions in ClinVar for c.221G > A variant in *SGCA* and c.2760dupC in *DYSF*; therefore, 80% of the discovered variants in our study were reported for the first time in the Iranian population. Although the prevalence of different types of LGMD differs by geographic location, LGMD2A is considered the most prevalent type of LGMD which represent 30–40% of autosomal recessive MDs (Chou et al., 1999; Dorobek et al., 2015). This is consistent with the number of diagnosed LGMD2A in our study (32%). In the Iranian population, a study by Fattahi Z et al has shown that 24% of neuromuscular disorders were diagnosed with pathogenic variants in the *CAPN3* (Fattahi et al., 2017). The most common countries with LGMD2A are Poland and Russia with 70 to 80% of LGMD patients (Dadali et al., 2010; Dorobek et al., 2015). Although C.550delA is the most common variant identified in many studies, our study revealed no variant in this genomic position. The exact prevalence of Dysferlinopathies is unclear worldwide, partly due to the absence of an international organization for the registry of these patients. Nevertheless, its frequency is estimated between 5% and 35% of LGMDs in a study by Taghizadeh et al. (2019). In some regions such as Maghreb, Israel, Saudi Arabia, Iran and India, dysferlinopathy is the second cause of LGMDs after calpainopathies. However, even in the populations with less consanguineous marriage like the USA, it accounts for 15% of recessive LGMD (Urtizberea et al., 2008). The prevalence of sarcoglycanopathies varies among different populations and it is generally estimated to be 9% (Kirschner & Lochmüller, 2011). In a study by Afagh Alavi et al on the Iranian population (Alavi et al., 2017), LGMD2E was suggested as the most common type of sarcoglycanopathies in Iran while type 2D has been suggested as the most common type worldwide (Frumento, 2019). The exon 2 deletion in the *SGCB* might be a common variant in the Iranian population due to multiple reports (Ghafouri‐Fard et al., 2017; Mojbafan et al., 2020; Taghizadeh et al., 2018) as in our study on the Iranian population.

## 4 METHODS AND MATERIALS

### 4.1 Ethical compliance

This study was approved by the Ethics Committee of Isfahan University of Medical Sciences, Isfahan, Iran and written informed consent was signed by all participating subjects or their first‐class families before sample collection.

### 4.2 Patients and clinical criteria

In this study, 26 patients from the Iranian population who had muscular abnormalities have referred to the hospital and were investigated over the course of 6 years. LGMD was suggested to be the first line diagnosis and patients were genetically examined to inspect the candidate genes for variants associated with muscular dystrophy. The patients were diagnosed with LGMD by clinicians based on clinical presentations, electromyography (EMG) and laboratory tests.

### 4.3 Genetics analysis

Peripheral blood samples were obtained from all subjects and genomic DNA was extracted using salting out protocol. The quality and quantity of DNA samples were assessed using a nanodrop2000 and agarose gel. All enriched DNA samples were subjected to whole‐exome sequencing on the Illumina Hiseq4000 platform with 100X depth of coverage and 101 bp paired‐end reads. Data analysis was performed using the GATK Best Practice pipeline using the Nextflow framework. Raw sequence data analysis, base calling and demultiplexing was performed and raw reads (fastq files) were aligned to the hg19 human reference genome (Genome Reference Consortium GRCh37) using BWA‐MEM tool. Picard tools were applied to collect alignment summary metrics and insert size metrics. Variant calling, SNP and indel extraction and filtering were performed using GATK tool algorithm (Genome Analysis Toolkit 2.4–4). Called variants were sorted to pathogenic or likely pathogenic on the basis of being homozygous missense, nonsense, frameshift, splice site, start codon change or intragenic indel with minimum allele frequency less than 1% acquired by population databases e.g., 1000 genomes, exome aggregation consortium (ExAC) and Genome Aggregation Database (genomAD). Meanwhile, intragenic deletion/duplications were included in the analysis. The suggestive causative variants were confirmed using conventional Sanger sequencing. In some patients, segregation analysis was performed to determine the most likely mode of inheritance considering the candidate gene and the disease phenotype.

### 4.4 Variant selection and sorting

All suspicious variants in candidate genes were sorted using bioinformatics tools based on their position in exon, intron and splice sites. The type of variant, amino acid substitution in missense variants and their proximity to the splice site are listed in respective tables. In order to determine the novelty of the variants, the ClinVar database and MutationTaster were explored for the candidate variants to check whether they have been previously reported in publications or as clinical evidence. Furthermore, genomAD was surfed not only to check the minimum allele frequency of the variants but also to make sure about the novelty of mutations. As a confirmation step, HGMD, Exac, 1000 genome and PubMed were surfed for the detected variant. The unanimous mRNA RefSeqs which were used to probe the variants in datasets and also to address in this paper are as follows: *CAPN3*: NM_000070.3, *DYSF*: NM_001130987.2, *SGCA*: NM_000023.4, *SGCB*: NM_000232.4, *SGCD*: NM_000337.5, *LAMA2*: NM_000426.3. As for the criteria to determine a novel variant, we considered 3 main features: being predicted as disease‐causing, the variant is not reported in the literature or evidence with any clinical association and the variant is not reported in wide genome studies as polymorphism at the time of the study. Variants were classified for pathogenicity based on the American College of Medical Genetics and Genomics (ACMG) guideline which is indicated in the tables (Richards et al., 2015). The GenBank reference sequence and version number for the genes are as follows: *CAPN3*: NG_008660.1, *DYSF*: NG_008694.1, *SGCA*: NG_008889.1, *SGCB*: NG_008891.1, *SGCD*: NG_008693.2, *LAMA2*: NG_008678.1.

### 4.5 Visualization

For secondary protein structure plotting, Plot Protein tool was executed in command line (Turner, 2013) using the corresponding isoform structure. The protein reference sequence accessions are as follows; *CAPN3*: NP_000061.1, *DYSF*: NP_001124459.1, *SGCA*: NP_000014.1, *SGCB*: NP_000223.1, *SGCD*: NP_000328.2 and *LAMA2*: NP_000417.3. Domains and motifs were extracted from different databases including human protein reference database (HPRD) (Peri et al., 2003), SMART (Letunic & Bork, 2018), and ensemble database. Post‐translation modifications were combined from HPRD and iPTMnet, GlyGen and PhosphoSite databases. Protein alignment was done using MUSCLE alignment tool (Edgar, 2004) using at least 5 different species in order to generate conservation plots in all panels of Figure 1.

## AUTHOR CONTRIBUTIONS

Conceptualization, Hamidreza Mianesaz; Data curation, Hamidreza Mianesaz and Safoura Ghalamkari; Formal analysis, Hamidreza Mianesaz and Safoura Ghalamkari; Funding acquisition, Maryam Sedghi; Investigation, Hamidreza Mianesaz, Mansoor Salehi and Mahdiyeh Behnam; Methodology, Hamidreza Mianesaz; Project administration, Behnaz Ansari; Final approval and resources, Majid Hosseinzadeh, Keivan Basiri and Majid Ghasemi; Software, Safoura Ghalamkari; Supervision, Mansoor Salehi and Maryam Sedghi; Validation, Maryam Sedghi and Behnaz Ansari; Writing – original draft, Hamidreza Mianesaz; Writing – review & editing, Safoura Ghalamkari and Maryam Sedghi. All authors have read and agreed to the published version of the manuscript.

## FUNDING INFORMATION

This work was funded by grant number 51079 from the deputy for research, Isfahan University of Medical Sciences, Isfahan, Iran.

## CONFLICT OF INTEREST

The authors declare that they have no known competing financial interests or personal relationships that could have appeared to influence the work reported in this paper.

## ETHICAL PUBLICATION STATEMENT

We confirm that we have read the Journal's position on issues involved in ethical publication and affirm that this report is consistent with those guidelines.

## ETHICS APPROVAL STATEMENT

This study was approved by the Ethics Committee of Isfahan University of Medical Sciences, Isfahan, Iran.

## PATIENT CONSENT STATEMENT

Written informed consent was signed by all participating subjects or their first‐class families before sample collection.

## Data Availability

The data that support the findings of this study are available on request from the corresponding author. The data are not publicly available due to ethical restrictions.
